# Femtosecond Laser-Engineered β-TCP Scaffolds: A Comparative Study of Green-Synthesized AgNPs vs. Ion Doping Against *S. aureus* for Bone Regeneration

**DOI:** 10.3390/ijms26104888

**Published:** 2025-05-20

**Authors:** Marco Oliveira, Liliya Angelova, Georgi Avdeev, Liliana Grenho, Maria Helena Fernandes, Albena Daskalova

**Affiliations:** 1 Institute of Electronics, Bulgarian Academy of Sciences, 72 Tsarigradsko Chaussee Blvd, 1784 Sofia, Bulgaria; moliveirall97@gmail.com (M.O.); lily1986@abv.bg (L.A.); 2 Institute of Physical Chemistry, Bulgarian Academy of Sciences, Akad. G. Bonchev Str., 1113 Sofia, Bulgaria; g_avdeev@abv.bg; 3 BoneLab—Laboratory for Bone Metabolism and Regeneration, Faculty of Dental Medicine, University of Porto, 4200-393 Porto, Portugal; lgrenho@fmd.up.pt (L.G.); mhfernandes@fmd.up.pt (M.H.F.); 4 LAQV/REQUIMTE–Associated Laboratory for Green Chemistry, Research Group “Materials for Sustainability and Wellbeing”, University of Porto, 4160-007 Porto, Portugal

**Keywords:** β-tricalcium phosphate, surface structuring, biocompatibility, femtosecond laser, silver nanoparticles, photoreduction, antibacterial potential

## Abstract

Implant-associated infections, particularly those linked to *Staphylococcus aureus* (*S. aureus*), continue to compromise the clinical success of β-tricalcium phosphate (β-TCP) implants despite their excellent biocompatibility and osteoconductivity. This investigation aims to tackle these challenges by integrating femtosecond (fs)-laser surface processing with two complementary strategies: ion doping and functionalization with green-synthesized silver nanoparticles (AgNPs). AgNPs were produced via fs-laser photoreduction using green tea leaf extract (GTLE), noted for its anti-inflammatory and antioxidant properties. Fs-laser processing was applied to modify β-TCP scaffolds by systematically varying scanning velocities, fluences, and patterns. Lower scanning velocities generated organized nanostructures with enhanced roughness and wettability, as confirmed by scanning electron microscopy (SEM), optical profilometry, and contact angle measurements, whereas higher laser energies induced significant phase transitions between hydroxyapatite (HA) and α-tricalcium phosphate (α-TCP), as revealed by X-ray diffraction (XRD). AgNP-functionalized scaffolds demonstrated markedly superior antibacterial activity against *S. aureus* compared to the ion-doped variants, attributed to the synergistic interplay of nanostructure-mediated surface disruption and AgNP-induced bactericidal mechanisms. Although ion-doped scaffolds exhibited limited direct antibacterial effects, they showed concentration-dependent activity in indirect assays, likely due to controlled ion release. Both strategies promoted osteogenic differentiation of human bone marrow mesenchymal stem cells (hBM-MSCs) under defined conditions, albeit with transient cytotoxicity at higher fluences and excessive ion doping. Overall, this approach holds promise for markedly improving antibacterial efficacy and osteogenic compatibility, potentially transforming bone regeneration therapies.

## 1. Introduction

Bacterial infections following bone substitute implantation remain one of the most daunting challenges in orthopedic and reconstructive surgery. Post-implant infections not only delay bone healing and can lead to non-union or implant failure but also impose a heavy economic burden on healthcare systems worldwide. For instance, prosthetic joint infections, primarily caused by pathogens such as *S. aureus* and *Staphylococcus epidermidis*, have been reported to occur in up to 2% of cases and are responsible for significant morbidity and revision surgeries [1,2]. These bacteria form resilient biofilms on implant surfaces—complex, self-produced extracellular polymeric matrices that protect bacterial cells from antibiotics and the host’s immune defenses [3].

Among the available bone substitutes, β-TCP has been widely adopted due to its excellent biocompatibility, osteoconductivity, and controlled biodegradability [4]. β-TCP provides an effective scaffold for new bone formation; however, its porous structure, which is beneficial for vascularization and cell infiltration, can also act as a niche for bacterial colonization and biofilm formation [5]. This paradox underscores the need for innovative strategies to enhance the antibacterial properties of β-TCP without impairing its osteogenic potential.

Various strategies have been explored to combat implant-associated infections. Traditional approaches, such as antibiotic loading and surface functionalization with antimicrobial peptides, often encounter challenges like antibiotic resistance and cytotoxicity [6,7]. To address these limitations, metal ion-doping has emerged as a promising alternative. Incorporating ions such as magnesium (Mg), strontium (Sr), silver (Ag), and copper (Cu) into scaffolds enables a controlled release of antibacterial agents while simultaneously enhancing osteogenesis.

Mg ions elevate the local pH and modulate membrane channels, creating an environment unfavorable for bacterial growth while promoting osteoblast differentiation [8]. Sr ions not only stimulate osteoblast activity [9] but also exhibit antibacterial properties by compromising bacterial membrane integrity and disrupting metabolic processes, reducing bacterial viability [10].

Ag ions are well known for their broad-spectrum antimicrobial effects. They interact with bacterial membranes, bind to intracellular proteins and DNA, and generate ROS, ultimately leading to bacterial cell death [11]. Similarly, Cu ions induce ROS production through Fenton-like reactions and compromise bacterial membranes, further enhancing antimicrobial efficacy [12].

While ion doping can provide prolonged antibacterial effects, another promising strategy is depositing AgNPs onto the scaffold surface. AgNPs exert a dual antibacterial action by releasing Ag⁺ ions and directly interacting with bacterial cells through physical disruption and oxidative stress [13]. Their potent antimicrobial properties are well documented; studies demonstrate that AgNPs induce oxidative stress, protein dysfunction, and damage to bacterial membranes and DNA, ultimately causing cell death [14,15]. Notably, the bactericidal efficiency of AgNPs may vary depending on the structural composition of the target bacteria. Gram-negative bacteria, which possess an outer membrane and a relatively thin peptidoglycan layer, often exhibit higher susceptibility to AgNP-induced membrane destabilization and nanoparticle internalization. In contrast, Gram-positive bacteria such as *S. aureus*, characterized by a thicker peptidoglycan matrix and absence of an outer membrane, can exhibit reduced nanoparticle penetration; yet, AgNPs have still shown potent activity against *S. aureus*, likely due to the generation of ROS, protein and DNA damage, and interference with metabolic pathways [16,17]. Moreover, the bactericidal efficacy of AgNPs is strongly influenced by their size and morphology, with smaller and spherical nanoparticles exhibiting enhanced interactions with bacterial cells due to facilitated membrane penetration and increased surface area-to-volume ratio, respectively [18]. Immobilizing AgNPs on surfaces enhances contact killing and minimizes the environmental risks associated with leaching [19]. Additionally, biologically synthesized AgNPs, especially those coated with antimicrobial metabolites, show higher internalization and increased ROS production compared to chemically stabilized nanoparticles, thereby enhancing their bactericidal activity [20].

However, at bactericidal concentrations (≥1–10 µg/mL), AgNPs can induce excessive ROS generation, mitochondrial dysfunction, and apoptosis in osteoblasts and mesenchymal stem cells, raising cytotoxicity concerns in bone-regenerative applications [21]. Moreover, clinical and environmental isolates carrying the sil operon have demonstrated resistance to AgNPs via efflux-pump upregulation, membrane-porin alterations, and biofilm-mediated Ag sequestration, which may compromise long-term efficacy [22]. To mitigate these challenges, AgNPs are frequently coated with ligands such as citrate or polyvinylpyrrolidone (PVP), which improve colloidal stability, modulate Ag⁺ release, and reduce oxidative stress, thereby preserving osteoblast viability without sacrificing antimicrobial potency [21]. Furthermore, the use of natural polyphenols in the synthesis of AgNPs offers a green approach that not only stabilizes the nanoparticles but also imparts antioxidant properties, potentially mitigating cytotoxic effects and improving biocompatibility [23].

A key advancement in AgNP synthesis is the use of fs-laser photoreduction in liquid media. Conventional chemical methods for AgNP synthesis often utilize strong reducing agents, such as sodium borohydride, N,N-dimethylformamide, and hydrazine [24,25], which raise concerns regarding biocompatibility. While less toxic alternatives like polyethylenimine (PEI) and citric acid have been employed, these agents can still exhibit cytotoxic effects under certain conditions. For instance, high-molecular-weight branched PEI has been associated with significant cytotoxicity in various cell types [26], and elevated concentrations of citric acid have demonstrated cytotoxicity in fibroblast cultures [27]. In contrast, fs-laser photoreduction is a rapid and environmentally friendly technique that eliminates the need for hazardous chemicals by using ultrashort laser pulses to reduce Ag⁺ ions directly in solution. This method is further enhanced by employing GTLE as a natural reducing and stabilizing agent. The polyphenols in GTLE accelerate the reduction of Ag⁺ to AgNPs while also adsorbing onto their surface to prevent aggregation, and their inherent antioxidant and anti-inflammatory properties may further support osteogenesis [28].

While the intrinsic chemical composition of a material plays a critical role in controlling bacterial and cellular adhesion, surface properties such as energy, wettability, and roughness are equally important in dictating cell–surface interactions [29,30]. In this context, fs-laser surface processing has emerged as a promising strategy to mitigate bacterial adhesion [31]. By delivering ultrashort laser pulses, fs-laser processing creates controlled micro- and nanoscale topographies on scaffold surfaces, all while minimizing thermal diffusion and avoiding structural damage. This precision allows for the optimization of wettability and roughness, ultimately reducing bacterial attachment or even endowing surfaces with bactericidal properties. Notably, fs-laser-induced nanostructures—such as nano-ripples and periodic surface patterns—have been shown to significantly diminish bacterial colonization and biofilm formation. For instance, nano-ripples have achieved antibacterial rates of up to 56% against *Escherichia coli* [32]. Similarly, laser-treated surfaces on materials like bioactive glass and metals have demonstrated complete bacterial rejection or marked reductions in biofilm biomass [33,34].

Our study investigates two distinct strategies for enhancing the antibacterial and bone-regenerative properties of β-TCP scaffolds. First, we employ fs-laser processing to modify the surface of both non-doped and ion-doped β-TCP scaffolds, generating controlled micro- and nanoscale topographies that may potentially deter bacterial adhesion while supporting the metabolic activity of human bone marrow-derived mesenchymal cells. Second, we utilize fs-laser photoreduction to synthesize AgNPs in liquid using GTLE from the Azorean Camellia sinensis as a green stabilizing agent, subsequently depositing these nanoparticles onto the laser-modified β-TCP surface. This dual approach enables a direct comparison between ion doping and AgNP functionalization in terms of their potential to enhance antimicrobial properties while assessing their effects on osteogenic response.

By integrating both physical (fs-laser-induced nanostructuring) and chemical (ion release or AgNPs) antibacterial strategies, this study aims to determine the choice of modification method and which modification may provide a more favorable balance between bacterial resistance and bone-regenerative potential. In summary, this work explores a multifunctional approach that combines fs-laser surface engineering with ion doping and green AgNP deposition, which could contribute to reducing implant-associated infections while promoting bone regeneration, thereby potentially improving the clinical outcomes in bone reconstruction therapies.

## 2. Results and Discussion

### 2.1. Morphological Characterization of the Laser-Treated β-TCP Scaffolds

In this section of the study, various techniques were employed to evaluate the surface characteristics of the fs-laser-treated β-TCP scaffolds. SEM was used for high-resolution imaging to examine the surface morphology. Three-dimensional optical profilometry was utilized to assess the surface’s roughness, providing detailed topographical measurements. XRD was employed to investigate the crystalline structure and phase composition of the scaffolds. Additionally, a Drop Shape Analyzer (DSA) was used to evaluate the wettability.

#### 2.1.1. SEM

**Figure 1:**
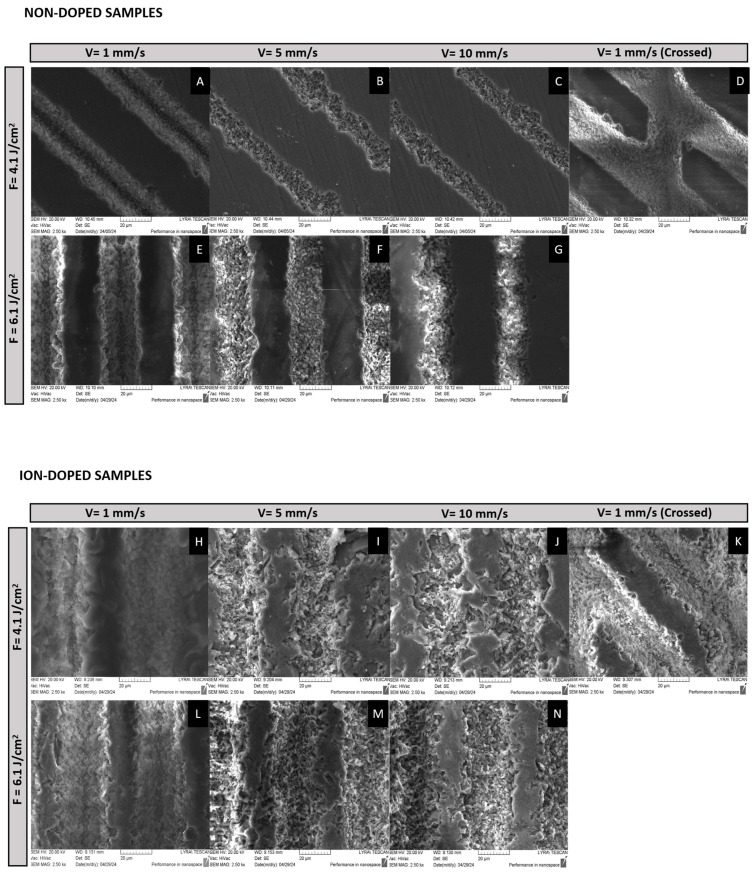
SEM micrographs illustrating the morphological alterations in ion-doped and non-doped β-TCP samples subjected to fs-laser treatment at various fluences (4.1 and 6.1 J/cm^2^), scanning velocities (1, 5, and 10 mm/s), and patterns (linear and crossed). For non-doped samples, the conditions are as follows: (**A**) V = 1 mm/s, F = 4.1 J/cm^2^; (**B**) V = 5 mm/s, F = 4.1 J/cm^2^; (**C**) V = 10 mm/s, F = 4.1 J/cm^2^; (**D**) V = 1 mm/s (Crossed), F = 4.1 J/cm^2^; (**E**) V = 1 mm/s, F = 6.1 J/cm^2^; (**F**) V = 5 mm/s, F = 6.1 J/cm^2^; (**G**) V = 10 mm/s, F = 6.1 J/cm^2^. For ion-doped samples, the conditions are as follows: (**H**) V = 1 mm/s, F = 4.1 J/cm^2^; (**I**) V = 5 mm/s, F = 4.1 J/cm^2^; (**J**) V = 10 mm/s, F = 4.1 J/cm^2^; (**K**) V = 1 mm/s (Crossed), F = 4.1 J/cm^2^; (**L**) V = 1 mm/s, F = 6.1 J/cm^2^; (**M**) V = 5 mm/s, F = 6.1 J/cm^2^; (**N**) V = 10 mm/s, F = 6.1 J/cm^2^. All micrographs were acquired with an acceleration of 20 kV and a magnification of 2500×.

The SEM results depicted in Figure 1 clearly show the impact of different laser conditions on the surface characteristics of both ion-doped and non-doped β-TCP samples. The analysis reveals that the scanning velocity plays a significant role in the material’s structural changes and that the laser structuring followed a similar pattern between the non-doped and ion-doped samples. At a scanning velocity of 1 mm/s, both tested fluences and in both non-doped and ion-doped samples, there is a noticeable improvement in the structural organization at the nanoscale (Figure 1A). This indicates a prolonged interaction of the laser with the material, enabling the formation of organized structures. These self-organized nanostructures are attributed to the cumulative energy input during laser treatment, which is consistent with the formation of laser-induced periodic surface structures (LIPSS) reported in previous studies [35].

In contrast, as the scanning velocity increases to 3.44 mm/s, a disruption of this structural organization is observed. This disruption results in the formation of nanoscale pores (Figure 1B), and this trend intensifies with further increases in velocity. At 10 mm/s, the material undergoes more pronounced structural disruptions, leading to the emergence of granular-like structures and additional nanopores (Figure 1C). The formation of these pores is related to the decrease in energy deposition as the velocity increases, leading to less controlled ablation and a shift towards more porous structures. Porosity is a beneficial characteristic for osteointegration [36], as it provides a scaffold for bone growth; however, this must be carefully balanced with the material’s antibacterial properties.

In an attempt to enhance interconnectivity between laser-modified lines, a cross-hatched pattern at a 45° angle was tested using a fluence of 4.1 J/cm^2^ while maintaining a scanning velocity of 1 mm/s. This approach resulted in the formation of well-defined rhombohedral structures, preserving a high degree of structural organization, even after repeated laser treatment (Figure 1D).

The effect of laser fluence on the resulting structures is also apparent in (Figure 1E–G,L,M). Higher energy levels, as expected, result in more intense material ablation, leading to disorganized structures with less defined boundaries. However, it is clear that the scanning velocity plays a more critical role in determining the material’s final structure, emphasizing the importance of careful control over scanning conditions to achieve the desired structural properties [37].

#### 2.1.2. Three-Dimensional Optical Profilometry

The three-dimensional optical profilometry revealed significant surface roughness variations across different laser conditions. Surface roughness (Sa) was influenced by both fluence and scanning velocity, with distinct trends for the non-doped and ion-doped samples.

**Figure 2:**
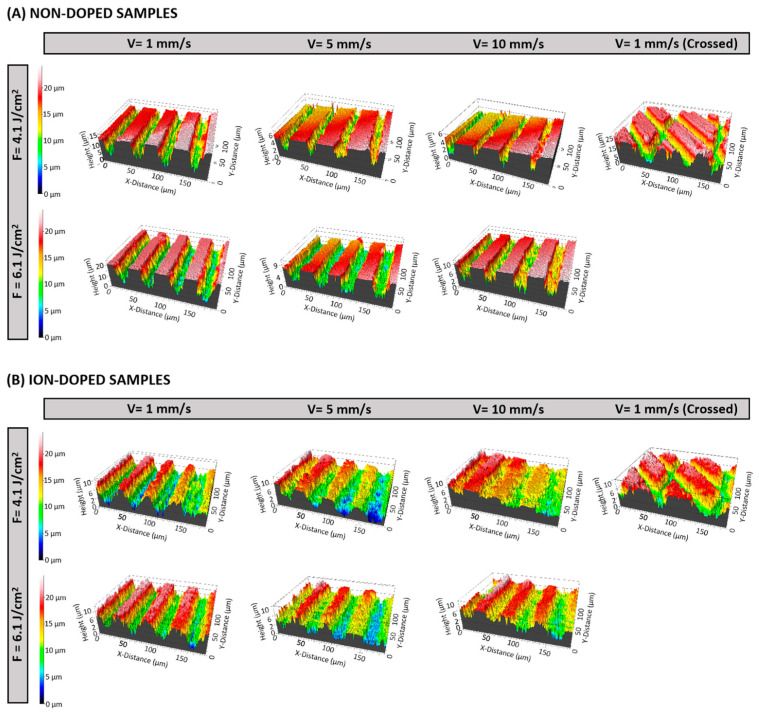
Three-dimensional optical profilometry images showing the variation in groove depth of β-TCP samples after fs-laser processing with varying fluences (4.1 and 6.1 J/cm^2^), scanning velocities (1, 5, and 10 mm/s), and irradiation patterns (linear and crossed), recorded at a magnification of 20×.

**Figure 3:**
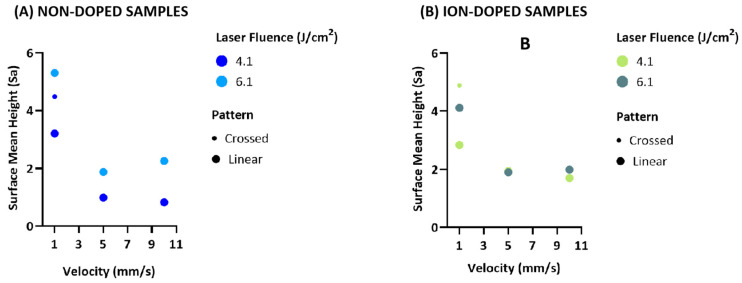
Multivariable bubble plot (**A**) and biplot of PCA analysis (**B**) illustrating the effects of fluence, scanning velocity, and patterns on the surface roughness parameter Sa.

For the non-doped samples, higher fluence (6.1 J/cm^2^) increased the roughness. At 4.1 J/cm^2^, Sa decreased with an increasing velocity, with 10 mm/s producing smoother surfaces than 5 mm/s. However, at 6.1 J/cm^2^, the roughness increased unexpectedly at 10 mm/s, suggesting a complex interaction between energy distribution and material response (Figure 2A and Figure 3A). This could be due to insufficient energy absorption at higher velocities, causing incomplete melting and irregular feature formation upon re-solidification. The introduction of crossed laser patterns (1 mm/s) also increased the roughness, likely due to overlapping laser spots generating additional surface features [38,39].

For ion-doped samples, a higher fluence similarly increased the roughness. At 5 mm/s, the Sa values for 4.1 J/cm^2^ and 6.1 J/cm^2^ were comparable, indicating that at this velocity, energy distribution might be more uniform, leading to similar surface textures. This suggests ion doping may stabilize the re-solidification process (Figure 2B and Figure 3B) [40]. As with non-doped samples, the crossed laser patterns resulted in the highest roughness, further confirming that overlapping laser paths can potentially disrupt uniform energy dissipation (Figure 2 and Figure 3).

These results show that velocity plays a key role in surface roughness, but its interaction with fluence becomes more complex at higher energy densities. The increased roughness at 10 mm/s under irradiation with 6.1 J/cm^2^ suggests that an insufficient interaction time may cause heterogeneous melting and re-solidification. The roughness enhancement with crossed patterns highlights the impact of overlapping ablation zones, increasing the chance of uneven melting and texturing due to multiple laser interactions in the same area [38,39].

#### 2.1.3. Wettability Analysis

**Figure 4:**
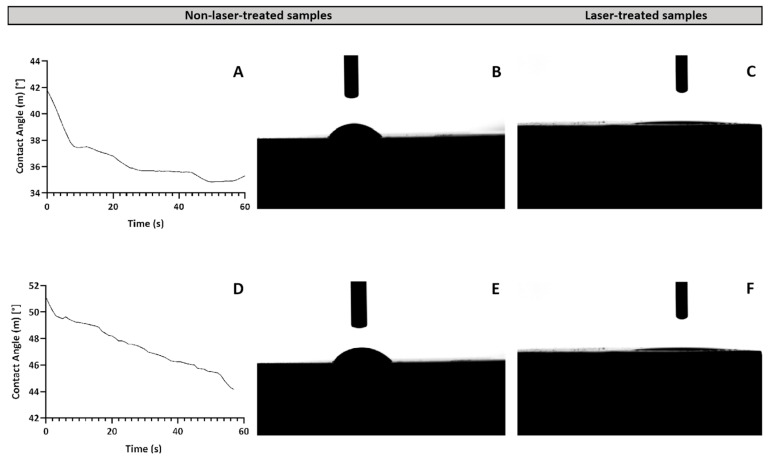
Panels (**A**–**C**) correspond to non-doped samples, and panels (**D**–**F**) represent ion-doped samples. (**A**,**D**) show graphs of the contact angle variation over time for non-laser-treated samples, indicating changes in wettability. (**B**,**E**) display representative images of water droplets on non-laser-treated samples, demonstrating their inherent wettability. (**C**,**F**) provide comparison images of water droplets on fs-laser-treated samples, highlighting the increased wettability of the treated surfaces.

The impact of laser treatments on surface wettability was assessed by measuring the water contact angles (WCAs) using the sessile drop method on both ion-doped and non-doped β-TCP samples. The results indicated a significant decrease in the WCA on laser-treated surfaces compared to the non-laser-treated samples (Figure 4A–C). In fact, the WCA reduction was so pronounced that it became impossible to measure the angle accurately, suggesting a transition of the surfaces from hydrophilic to superwetting after laser ablation (Figure 4C).

This enhancement in hydrophilicity can be explained by the Wenzel model, which posits that an increased surface roughness increases the actual contact area between the surface and the liquid, thus improving wettability and lowering the contact angle. The observed transition to superwetting surfaces is particularly significant for biomedical applications, where it could improve protein adhesion, thereby promoting more efficient tissue integration and better incorporation of implants [41].

#### 2.1.4. XRD Analysis

**Figure 5:**
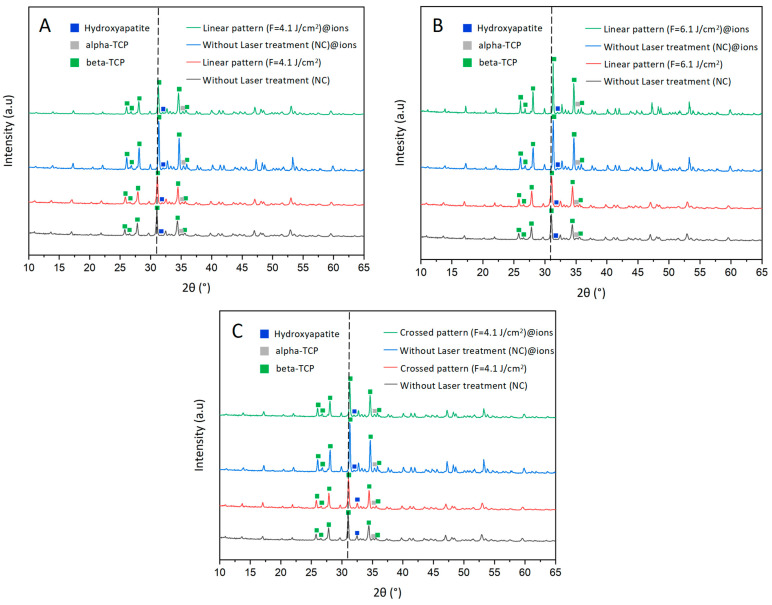
XRD spectra of β-TCP samples under different conditions, comparing laser-treated and untreated surfaces. The spectra include ion-doped and non-doped samples subjected to laser treatment with different patterns and fluences: (**A**) linear pattern at 4.1 J/cm^2^, (**B**) linear pattern at 6.1 J/cm^2^, and (**C**) crossed pattern at 4.1 J/cm^2^. For each condition, the corresponding non-laser-treated samples are also shown as negative controls (NC). Major diffraction peaks corresponding to β-TCP, α-TCP, and HA are labeled in the figures. A black dashed line is included to assist in visualizing the slight rightward shift observed in the ion-doped samples.

X-ray diffraction (XRD) was employed to analyze the crystalline phase composition of the produced tricalcium phosphate scaffolds and investigate the structural modifications induced by ion doping and laser processing. This technique enables the identification of phase transformations among HA, β-TCP, and α-TCP, which are critical in determining the bioactivity, resorption rate, and mechanical properties of bone substitutes [42]. XRD analysis confirms that in the non-doped samples, β-TCP is the predominant phase, with minor contributions from HA and α-TCP. Notably, a slight shift in the characteristic β-TCP peaks (2θ ≈ 28°, 31°, and 34.5°) to the right in the ion-doped samples suggests that metallic ions are successfully incorporated into the TCP lattice, replacing Ca^2^⁺ and inducing lattice distortions that shift the peaks toward higher angles (Figure 5) [43].

**Figure 6:**
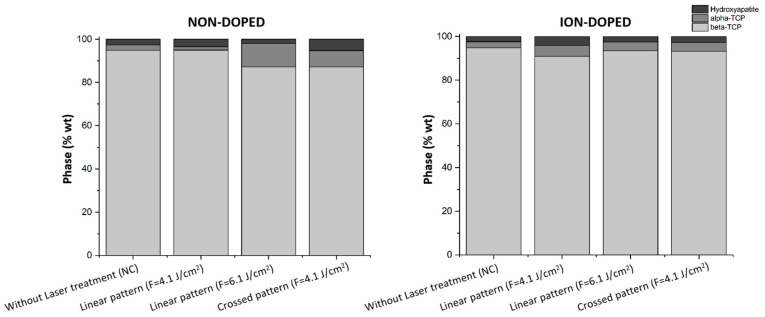
Phase composition of β-TCP samples under different conditions, comparing laser-treated and untreated surfaces. The bar charts represent the phase distribution (wt%) of β-TCP, α-TCP, and HA in non-doped (**left**) and ion-doped (**right**) samples. Each graph includes negative controls (NC) without laser treatment and samples subjected to laser processing with a linear pattern at F = 4.1 J/cm^2^, a linear pattern at F = 6.1 J/cm^2^, and a crossed pattern at F = 4.1 J/cm^2^.

In the non-doped samples, increasing the laser fluence to 6.1 J/cm^2^ significantly enhances phase transformation (Figure 6), resulting in a reduction of β-TCP and the emergence of α-TCP as a secondary phase. This transformation is attributed to localized heating, as α-TCP represents the high-temperature polymorph of tricalcium phosphate and typically forms above 1125 °C [44]. In contrast, when a lower fluence of 4.1 J/cm^2^ is applied in a crossed pattern—implying repetitive laser exposure—the β-TCP phase remains relatively unchanged. However, the phase composition shifts notably, with nearly equal proportions of HA and α-TCP emerging. These findings suggest that while high fluences predominantly favor the formation of α-TCP, lower fluences combined with repeated laser exposure promote the transition towards both HA and α-TCP, highlighting the influence of cumulative thermal cycles on the material’s phase composition.

Interestingly, ion doping appears to counteract these laser-induced transformations by stabilizing β-TCP and suppressing α-TCP formation. As illustrated in Figure 6, the doped samples retain a higher proportion of β-TCP, even under elevated fluences, indicating that dopant ions contribute to lattice stabilization and inhibit excessive phase conversion [45,46]. In some cases, a slight increase in HA content is also observed, which may enhance bioactivity and osteoconductivity [47,48]. The formation of HA under these conditions may stem from localized recrystallization, driven by moderate thermal effects. Repetitive laser exposure can promote ionic diffusion and structural reorganization, favoring HA formation, especially in hydrated or hydroxylated surface regions. As HA is thermodynamically stable at physiological conditions, even mild heating can facilitate its development as a secondary phase [49].

A moderate laser fluence of 4.1 J/cm^2^ emerges as an optimal condition, striking a balance between inducing beneficial structural modifications and maintaining phase stability, particularly in doped samples. This trend is evident in both Figure 5, where peak shifts and secondary phase formation are less pronounced, and Figure 6, where β-TCP remains the dominant phase. Preserving a stable β-TCP/HA ratio is highly desirable, as these phases are known to support enhanced resorption rates and provide mechanical properties suitable for bone regeneration applications.

### 2.2. In Vitro Cytocompatibility

In this phase of the study, the cytocompatibility of fs-laser-treated surfaces was evaluated by assessing the hBM-MCs growth using the resazurin assay. The tested samples were those with the most promising nanoscale structural organization: linear pattern (F = 4.1 J/cm^2^), linear pattern (F = 4.1 J/cm^2^)@ions, linear pattern (F = 6.1 J/cm^2^), linear pattern (F = 6.1 J/cm^2^)@ions, linear pattern (F = 4.1 J/cm^2^)@AgNPs, crossed pattern (F = 4.1 J/cm^2^), and crossed pattern (F = 4.1 J/cm^2^)@ions. Their effects on cell proliferation and viability were compared to an untreated control to determine the most cytocompatible surface modifications.

**Figure 7:**
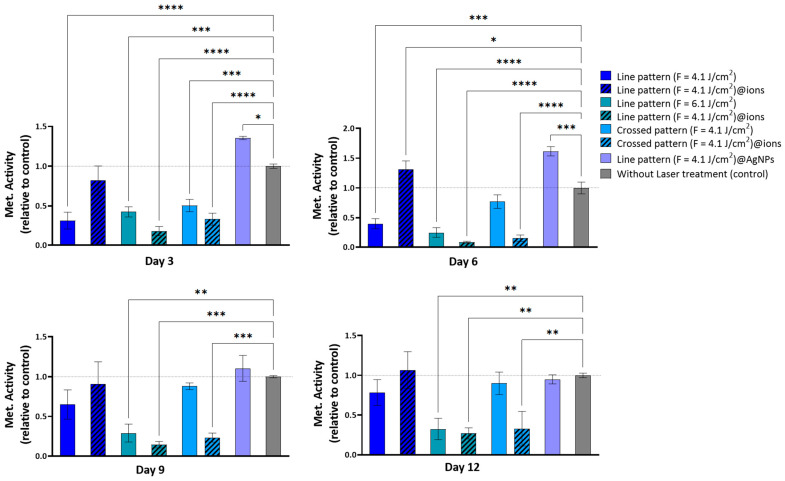
Metabolic activity of hBM-MSCs cultured over the fs-laser-treated β-TCP scaffolds for periods up to 12 days. Results are presented relative to the untreated samples (control, set up at 1.0, dotted line). Statistically different from control: * *p* < 0.05, ** *p* < 0.01, *** *p* < 0.001, and **** *p* < 0.0001.

Figure 7 illustrates the intricate relationship between surface modifications—determined by laser fluence, patterning, and doping (non-doped, ion-doped, and AgNPs-doped)—and cellular responses. Notably, the linear pattern at a higher fluence (fluence = 6.1 J/cm^2^) exhibited a consistent reduction in metabolic activity across all time points. By Day 3, this condition led to a significant decrease in cell viability by approximately 57% (*p* < 0.01), which further declined to nearly 75% (*p* < 0.001) by Day 9 (Figure 7). This cytotoxic effect may be attributed to increased surface roughness and wettability, potentially enhancing the release of phosphate and calcium ions. Excessive cytosolic calcium ion accumulation can be detrimental, potentially triggering cell death [50,51].

Conversely, the linear pattern (fluence = 4.1 J/cm^2^) and the crossed pattern (fluence = 4.1 J/cm^2^) supported a gradual increase in metabolic activity over time, despite initially reduced values. For the linear pattern (fluence = 4.1 J/cm^2^), metabolic activity on Day 3 was approximately 32% of the control *p* < 0.001), but progressively increased to about 65% by Day 9 and 80% by Day 12 (Figure 7). Similarly, the crossed pattern (fluence = 4.1 J/cm^2^) followed a comparable trend, with initial metabolic activity around 49% of the control on Day 3 (*p* < 0.01), becoming non-significant by Day 6 and continuing to improve thereafter (Figure 7). These findings suggest that, while these surface modifications initially impose cellular stress, they do not induce long-term cytotoxicity; rather, cells adapt to the modified environment, leading to progressive viability recovery.

The incorporation of ionic doping had a marked influence on cytocompatibility outcomes. Surfaces created at higher fluences—such as the linear pattern (fluence = 6.1 J/cm^2^)@ions and the crossed pattern (fluence = 4.1 J/cm^2^)@ions—exhibited a pronounced reduction in metabolic activity at all time points. This effect may be attributed to the enhanced roughness and hydrophilicity of these surfaces, which could lead to increased solubility and subsequent ion release. Although elements like Sr, Mg, Zn, and Cu are known to support bone cell function, their excessive presence can have deleterious effects.

Sr and Mg, for instance, influence bone metabolism in a concentration-dependent manner. While low levels of strontium activate osteogenic pathways such as AMPK/mTOR, excessive concentrations disrupt HA formation, impairing mineralization. Similarly, Mg modulates calcium signaling and osteoblast differentiation, but its excessive presence interferes with bone matrix deposition [52,53,54]. 

Likewise, zinc and Cu play essential roles in bone homeostasis but become cytotoxic at high concentrations. Zinc, when present in excess, leads to a marked reduction in cell viability, with studies indicating a 50% viability decrease at an alloy extract concentration of 0.75 mg/mL, though recovery was observed by Days 3 and 7. Meanwhile, elevated Cu levels induce severe cytotoxic effects, including granular basophilia, lacunar detachment, and necrosis [55,56].

However, the linear pattern (fluence = 4.1 J/cm^2^)@ions resulted in a notable increase in metabolic activity, peaking on Day 6 with a 131% (*p* < 0.05) increase, followed by a minor decrease on Day 9 and a slight resurgence on Day 12. This trend closely mirrors the effect observed with AgNPs, suggesting a shared mechanism underlying these metabolic responses. The introduction of AgNPs into the linear pattern (fluence = 4.1 J/cm^2^) demonstrated a remarkable ability to enhance cell viability. This effect was particularly evident on Day 6, where metabolic activity reached 162% of the control (*p* < 0.01) (Figure 7). These findings suggest that both AgNPs and ionic doping may play a crucial role in mitigating the initial cytotoxic effects of surface modifications while fostering cellular adaptation.

**Figure 8:**
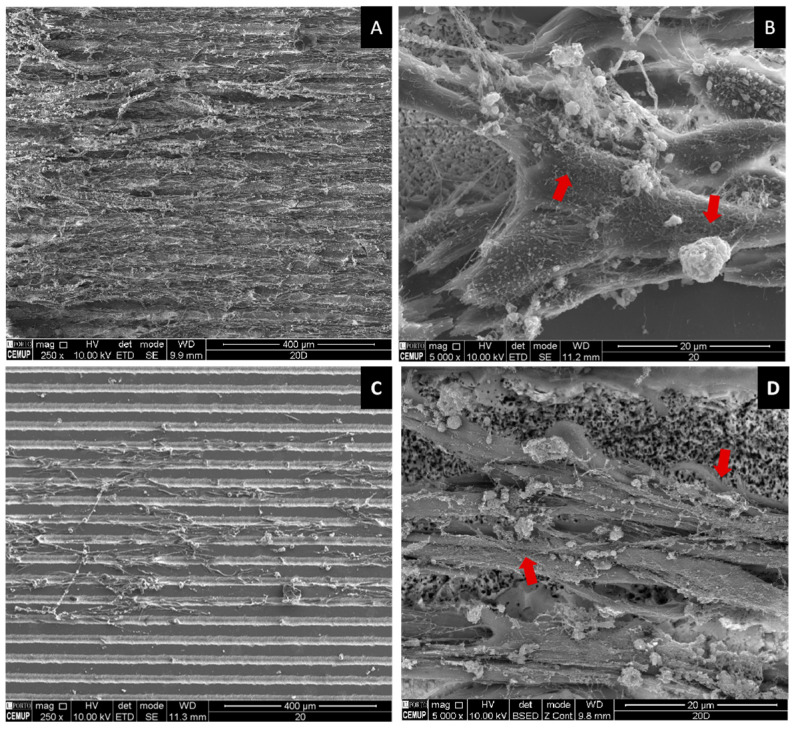
SEM representative images of human hBM-MSCs cultured on fs-laser-treated β-TCP scaffolds for 12 days under the linear pattern condition (fluence = 4.1 J/cm^2^). Low-magnification images (250×) are shown in panels (**A**,**C**), while high-magnification images (5000×) are presented in panels (**B,D**). Panels (**A**,**B**) depict non-doped samples, whereas panels (**C**,**D**) illustrate ion-doped samples. Red arrows highlight examples of mineralized deposits.

Moreover, SEM analysis revealed that the cells aligned along the surface patterns (Figure 8 A–C) and exhibited mineralized matrix formations and vesicular bodies (Figure 8B–D). The synergistic effect between AgNPs and potentially adhered phenolic compounds, which possess antioxidant and anti-inflammatory properties, likely contributed to these favorable outcomes by mitigating oxidative stress and fostering an optimal environment for cellular proliferation [57]. However, a decline in viability/proliferation was noted beyond Day 6, as also observed in the linear pattern (fluence = 4.1 J/cm^2^)@ions condition. This reduction may be attributed to the heightened initial stimulation of cell growth by AgNPs, leading to earlier confluence and subsequent contact inhibition, a well-documented mechanism in rapidly proliferating cultures [58,59]. Supporting this hypothesis, viability levels on Days 9 and 12 were comparable to those observed in the linear pattern (fluence = 4.1 J/cm^2^), crossed pattern (fluence = 4.1 J/cm^2^), and untreated conditions, suggesting that the observed reduction is unlikely due to intrinsic AgNP cytotoxicity.

Another plausible explanation is the differentiation of cells over time. A metabolomic analysis by Bispo et al. [60] identified significant shifts in over 30 metabolites during a 21-day osteogenic differentiation period, highlighting the dynamic nature of this process. Given that metabolic fluctuations accompany differentiation, these changes could be influencing the viability trends detected via the resazurin assay. SEM imaging further corroborates this notion, as cells on the scaffolds not only aligned with the surface topography but also exhibited mineralized matrix formations and vesicular bodies, indicative of early bone tissue differentiation and matrix deposition [61].

### 2.3. Antibacterial Activity

The antibacterial potential of β-TCP scaffolds was evaluated against *S. aureus* (ATCC 25923) to assess their potential for infection prevention in biomedical applications. Bacterial suspensions were prepared in tryptic soy broth (TSB) at 37 °C until reaching a concentration of 10^6^ CFU/mL. A direct antibacterial assay was performed by incubating the scaffolds with *S. aureus* suspensions for 24 h, followed by a viability assessment through fluorescence measurements using a resazurin-based assay. Additionally, an indirect assay was conducted by exposing *S. aureus* to scaffold-derived extracts at varying concentrations (10%, 50%, and 90%) to determine the antimicrobial effects of soluble factors. Fluorescence intensity data were normalized to untreated β-TCP scaffolds, which served as the control group.

**Figure 9:**
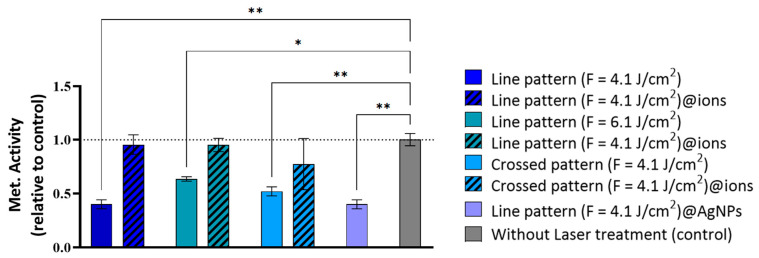
Antibacterial activity of the fs-laser-treated non-doped, ion-doped, and AgNPs-doped β-TCP scaffolds against *S. aureus* grown directly on the scaffolds’ surfaces. Results are presented relative to the untreated samples (control, set up at 1.0, dotted line). Statistically different from control: * *p* < 0.05 and ** *p* < 0.01.

As depicted in Figure 9, fs-laser-treated β-TCP scaffolds significantly reduced the sessile bacterial population. The direct resazurin assay revealed the most pronounced antibacterial effect with the linear pattern (F = 4.1 J/cm^2^), particularly when combined with AgNPs, leading to bacterial viability reductions of 50% (*p* < 0.01) and 60% (*p* < 0.01), respectively (Figure 9). The linear pattern with higher laser fluence (F = 6.1 J/cm^3^) achieved a 36% viability reduction (*p* < 0.05), while the crossed pattern (F = 4.1 J/cm^2^) resulted in a 48% reduction (*p* < 0.01), albeit this was less effective than the linear pattern (F = 4.1 J/cm^2^) (Figure 9). These observations are likely attributable to the formation of nanoscale, self-organized topographical features analogous to LIPSS, which disrupt bacterial colonization [62]. Moreover, the incorporation of AgNPs confers synergistic antibacterial and anti-adhesive properties. The primary mechanism appears to be the modification of the surface microenvironment, rendering it inhospitable to bacterial adhesion rather than solely relying on the direct bactericidal effects of AgNPs. Although higher fluence treatments and crossed patterning also reduced bacterial adhesion, their efficacy was inferior to that achieved with the linear pattern in conjunction with AgNPs, underscoring the critical role of AgNPs in inhibiting bacterial attachment.

Beyond the surface modifications induced by laser ablation, quantitative proteomic studies by Zhang et al. suggested that AgNPs and Ag ions interfere with bacterial chemotaxis [63]. Li et al. further reported the inhibition of several enzymes and ATP-binding proteins in *S. aureus* [64], while Mirzajani et al. observed the suppression of membrane proteins involved in electron transport in *Bacillus thuringiensis* [65]. These findings support the hypothesis that AgNPs do not necessarily need to be internalized by bacterial cells to exert their antimicrobial effects; instead, they may disrupt the chemotactic signaling pathways or induce metabolic deregulation through interactions with the bacterial membrane. In addition to the intrinsic antibacterial properties of AgNPs, the incorporation of polyphenols from GTLE onto their surface may enhance antibacterial activity, particularly against Gram-positive bacteria such as *S. aureus*. Polyphenolic compounds, including catechins, exhibit antimicrobial properties, such as the disruption of bacterial membrane integrity, inhibition of key enzymatic functions, and modulation of oxidative stress. Specifically, epigallocatechin gallate has been shown to inhibit DNA gyrase in *S. aureus*, thereby disrupting essential processes of bacterial replication and transcription [66]. The synergistic interaction between AgNPs and polyphenols likely contributes to the enhanced antibacterial efficacy observed. However, further investigation is needed to fully elucidate these mechanisms.

**Figure 10:**
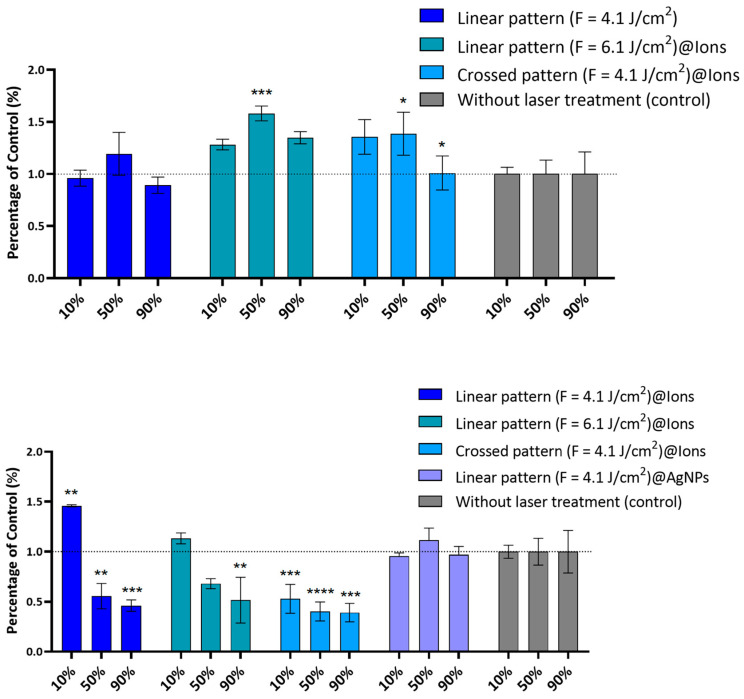
Antibacterial activity of the fs-laser-treated non-doped, ion-doped, and AgNPs-doped β-TCP scaffold-derived extracts against *S. aureus*. The upper panel shows non-doped samples, while the lower panel displays ion-doped samples. Results are presented relative to the untreated samples (control, set up at 1.0, dotted line). Statistically different from control: * *p* < 0.05, ** *p* < 0.01, *** *p* < 0.001 and **** *p* < 0.0001.

In contrast, the ion-doped samples exhibited no significant antibacterial effect in the direct assay (Figure 10) when bacteria were cultivated directly on β-TCP surfaces. However, in the indirect assay, scaffold-derived extracts demonstrated a concentration-dependent antibacterial effect, particularly in the linear pattern (F = 4.1 J/cm^2^)@ions, reducing bacterial metabolism by 44% (*p* < 0.01) and 54% (*p* < 0.001) at extract concentrations of 50% and 90%, respectively. The crossed pattern (F = 4.1 J/cm^2^)@ions exhibited bacterial metabolic reductions of 60% (*p* < 0.0001) and 61% (*p* < 0.001) at the same extract concentrations, respectively. Notably, in this assay, neither the non-doped samples nor those incorporating AgNPs displayed antibacterial activity. This aligns with our earlier findings, suggesting that AgNPs remain surface-bound and exert their antimicrobial effect primarily at the interface, as observed in the direct assay. Conversely, ion-doped samples appear to facilitate a substantial ion release, contributing to a notable antibacterial effect that is not as evident in the direct assay.

**Figure 11:**
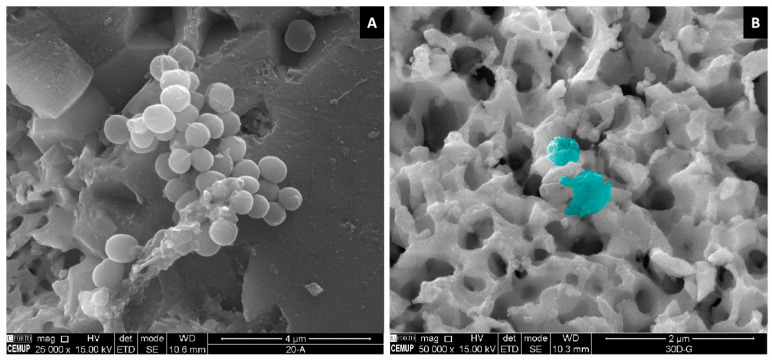
Representative SEM micrographs of *S. aureus* adhesion on β-TCP surfaces. *S. aureus* cells are observed on the non-laser-treated region at 25,000× magnification (**A**). In contrast, at 50,000× magnification, bacteria on the bottom of the laser-treated region (**B**) exhibit a disrupted morphology (blue).

The SEM images further demonstrated a higher accumulation of bacteria in the untreated regions compared to the laser-treated areas. Additionally, some bacteria in the laser-treated region exhibited disrupted membrane morphologies (Figure 11).

## 3. Materials and Methods

### 3.1. Chemicals

Calcium nitrate tetrahydrate [Ca(NO_3_)_2_·4H_2_O], strontium nitrate [Sr(NO_3_)_2_], copper(II) nitrate hemi-(pentahydrate) [Cu(NO_3_)_2_·2.5H_2_O], silver nitrate [AgNO_3_], and magnesium nitrate hexahydrate [Mg(NO_3_)_2_·6H_2_O] were used as precursors in the synthesis of ion-doped β-TCP scaffolds, all supplied by Merck (Darmstadt, Germany). Ammonium phosphate dibasic [(NH_4_)_2_HPO_4_], used as the phosphate source, was also obtained from Merck. Darvan C*^®^* dispersant (R.T. Vanderbilt Co., Norwalk, CT, USA) was used in the slurry preparation for casting. For the green synthesis of AgNPs, AgNO_3_ served as the metallic precursor, and GTLE was prepared in-house from dried green tea leaves sourced from Gorreana Tea Plantations (São Miguel, Azores, Portugal). For the cell culture experiments, alpha-MEM, fetal bovine serum (FBS), penicillin, streptomycin, and amphotericin B were purchased from Gibco (Bridgewater, NJ, USA). Resazurin sodium salt, used in the viability assays, was obtained from Sigma-Aldrich (St. Louis, MO, USA). For the antibacterial assays, *S. aureus* was cultured in tryptic soy broth (TSB; Liofilchem, Roseto degli Abruzzi, Italy). All chemicals were used as received, without further purification.

### 3.2. Fabrication of β-TCP Scaffolds

The fabrication of β-TCP disks followed a co-precipitation method previously established by the research group [67]. The process began with dissolving calcium nitrate tetrahydrate in distilled water inside a 6 L double-walled glass reactor. To obtain doped β-TCP, predetermined amounts of metal salts, specificallySr nitrate, copper (II) nitrate hemi-(pentahydrate), silver nitrate, and magnesium nitrate hexahydrate, were introduced into the reactor. These salts were added in quantities, ensuring the final stoichiometric composition of the doped materials: 2% Mg, 2% Sr, 0.1% Ag, and 0.1% Cu, expressed as molar percentages.

Subsequently, a solution of ammonium phosphate dibasic was gradually introduced into the reactor at a controlled flow rate of 10 mL/min using a peristaltic pump. Throughout the reaction, the temperature was maintained at 31 °C, while pH adjustments were carried out using ammonia to stabilize the system at pH 6.7 for undoped β-TCP and pH 7.2 for metal-doped variants. The reaction mixture was continuously stirred under mechanical agitation, and, following the complete addition of the phosphate solution, the system was left to mature for 20 h while agitation continued.

Once the maturation period was complete, the resulting slurry was filtered and dried. The dried material then underwent a three-step calcination process before being finely milled. The resulting powder was subsequently mixed with distilled water and Darvan C^®^ dispersant in a ball-milling system to create a homogeneous slurry. This slurry was then cast into molds and allowed to dry at 40 °C overnight. Finally, the dried material was subjected to a sintering process, yielding β-TCP disks with a diameter of 1 cm.

### 3.3. Laser Processing of β-TCP Scaffolds

**Figure 12:**
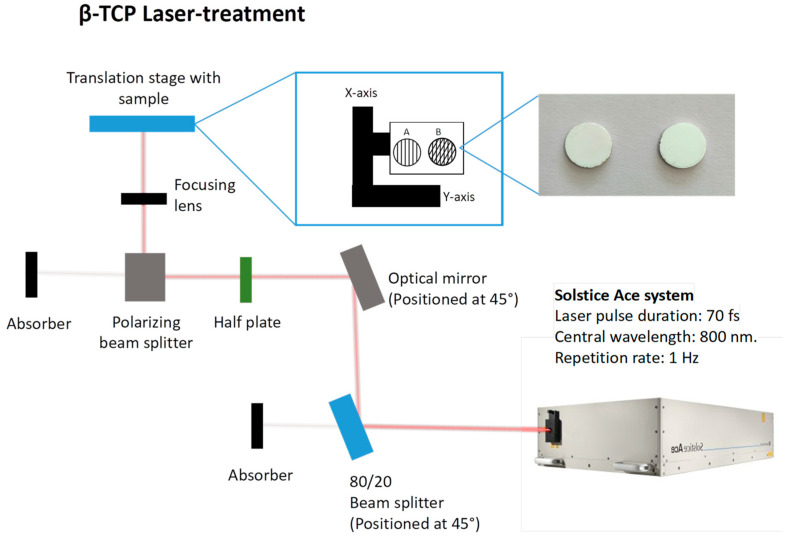
Schematic representation of the laser setup employed for the surface modification of β-TCP samples.

Laser ablation was carried out, as illustrated in Figure 12, using a Solstice Ace system (Spectra-Physics, Milpitas, CA, USA), which operates at a pulse duration of 70 femtoseconds and a central wavelength of 800 nm. The samples were secured to a glass slide with double-sided adhesive tape and placed on a two-axis motorized translation stage (Thorlabs, Newton, NJ, USA), which was controlled by Kinesis^®^ Software version 1.14.45 (Thorlabs, Newton, NJ, USA). The laser beam passed through an 80/20 beam splitter, with 80% of the light directed toward the sample and 20% used for diagnostic purposes, such as the pulse width and beam profile measurements. Energy variations in the laser radiation were achieved through a polarizing beam splitter coupled with a half-wave plate. The laser beam was afterwards focused onto the sample using a 200 mm focal length achromatic convex lens, resulting in a focal spot of 25 μm in diameter (Figure 1). Ablation was performed at a fixed repetition rate of 1 kHz for all samples. The disk surfaces were scanned in a raster pattern at various speeds (1, 5, and 10 mm/s) and fluences (4.1 and 6.1 J/cm^2^). The resulting ablation patterns, shown in Figure 1, featured either parallel lines (linear pattern) or a grid-like arrangement (crossed pattern) with orthogonal lines intersecting at 45°. A hatch distance of 50 μm was used for all patterns, except for the grid formations, where the hatch distance for the intersecting lines was set at 100 μm.

### 3.4. Morphological Characterization of the Laser-Treated β-TCP Scaffolds

#### 3.4.1. SEM

The laser-treated matrices were analyzed with SEM using two different systems: the “Lyra” from Tescan Orsay Holding (Brno-Kohoutovice, Czech Republic) and the TM4000 from Hitachi High-Tech Europe (Krefeld, Germany). To improve the visibility of the samples, a thin gold layer, approximately 4 nm thick, was applied by sputtering before imaging. The SEM observations were carried out at an accelerating voltage of 20 kV.

#### 3.4.2. Three-Dimensional Optical Profilometry

Surface morphological alterations caused by different laser pulse applications were evaluated using a 3D optical profilometry system (Leica DCM 3D, Berlin, Germany). The images were captured at a 20× magnification in true color. The surface roughness of the treated areas was quantified based on the ISO 4287 standard [68], utilizing the arithmetical mean height (Sa) as the measurement parameter. The 3D optical data were analyzed with ProfilmOnline software (www.profilmonline.com, accessed on 2 June 2024).

#### 3.4.3. Wettability Analysis

The effect of the laser treatments on surface wettability was assessed using a DSA100 Drop Shape Analyzer (KRÜSS GmbH, Hamburg, Germany), an optical system designed for video-based contact angle measurements. Both untreated and laser-modified surfaces were tested with distilled water (high polarity). The contact angles were measured at room temperature using the sessile drop method with 2 μL droplets. At least three droplets were applied to each sample type. The evolution of each droplet was observed over a period of three minutes, with measurements taken every second during the first minute and once every minute thereafter. The contact angles were calculated using ADVANCE software version 1.7.2.2 (KRÜSS, Hamburg, Germany), fitting the droplet profiles to the Young–Laplace equation.

#### 3.4.4. XRD Analysis

XRD was employed to quantify the crystalline phase composition of β-TCP, α-TCP, and HA in the laser-processed scaffolds. Data acquisition was performed using an EMPYREAN diffractometer (Malvern Panalytical, Almelo, The Netherlands) equipped with a position-sensitive PIXcel detector. Measurements were conducted in a Theta/Theta configuration over a 2θ range of 4.03° to 79.98° at 25 °C, utilizing Cu-Kα1 radiation (λ = 1.5406 Å), an X-ray tube operating at 40 kV and 30 mA, and a fixed divergence slit of 0.5°. The scan was carried out in continuous mode with a step size of 0.053° 2θ and a scan time of 84.4 s per step, ensuring high-resolution phase identification and quantification. Phase matching was completed using the Rietveld refinement with the Profex^®^ (version 5.0) software.

### 3.5. Green Laser-Assisted Synthesis of AgNPs and Deposition in β-TCP Pellets

The synthesis process followed a previously established method utilized by our research group [69,70]. Briefly, AgNPs were synthesized via fs-laser-assisted photoreduction, employing silver nitrate as the precursor and GTLE as the reducing and stabilizing agent, owing to the polyphenol content.

GTLE was prepared by boiling 8 g of dried green tea leaves in 50 mL of ultrapure water for 5 min, followed by filtration and storage at 4 °C. AgNP synthesis was conducted by mixing 400 µL of a 10 mM AgNO_3_ solution with 4 mL of a 10% (*v*/*v*) GTLE solution, followed by fs-laser irradiation at a fluence of 8.1 J/cm^2^ for 8 min.

Following irradiation, AgNPs were purified by centrifugation (4000 rpm, 10 min), washed with deionized water, and resuspended in water to yield a concentration of 2 mM. The obtained AgNPs, with sizes of approximately 15 nm at this concentration, were subsequently deposited onto laser-treated β-TCP scaffolds (1 mm/s, 4.1 J/cm^2^) and dried at room temperature for 8 h.

### 3.6. Biological Activity

The experimental conditions selected for the in vitro biological assays are represented in the following Table 1.

#### 3.6.1. In Vitro Cytocompatibility

##### Cell Culture Conditions

The biocompatibility of the materials was assessed in vitro following the ISO 10993 guidelines [71] using commercially available hBM-MC cells. These cells were maintained in alpha minimum essential medium (α-MEM) supplemented with 10% FBS, along with 100 IU/mL of penicillin, 100 µg/mL of streptomycin, and 2.5 µg/mL of amphotericin B. All culture reagents were obtained from Gibco (Bridgewater, NJ, USA). The cells were incubated at 37 °C in a humidified environment containing 5% CO_2_.

##### Direct Cytocompatibility Assay

To examine the ability of hBM-MCs to proliferate directly on β-TCP scaffolds, the cells were seeded onto β-TCP pellets at a density of 3 × 10^5^ cells/cm^2^ and cultured in a complete medium for 12 days within 24-well plates. The cell metabolic activity on the β-TCP samples was analyzed using the resazurin assay at four time points: Days 3, 6, 9, and 12. Before the measurements, all samples were transferred to new well plates and incubated for 3 h in a 10% resazurin solution (resazurin sodium salt, 0.1 mg/mL, Sigma-Aldrich, St. Louis, MO, USA) prepared in a complete medium at 37 °C. Fluorescence readings (excitation at 530 nm, emission at 590 nm) were obtained using a Synergy HT microplate reader (Biotek, Winooski, VT, USA) with Gen5 1.09 Data Analysis Software. The data were normalized relative to the untreated β-TCP scaffolds, which served as the control.

### 3.7. Antibacterial Activity

#### 3.7.1. Bacterial Culture Conditions

The antibacterial properties of the β-TCP ceramic scaffolds were evaluated using *S. aureus* (ATCC 25923) [72]. To prepare the bacterial suspensions, *S. aureus* was cultured in TSB (Liofilchem, Roseto degli Abruzzi, Italy) at 37 °C until reaching a density of 10^6^ colony-forming units (CFUs) per milliliter.

#### 3.7.2. Antibacterial Direct Assay

To evaluate the antibacterial activity of the β-TCP scaffolds, their upper surfaces were exposed to 1 mL of a prepared *S. aureus* suspension in 24-well plates and incubated at 37 °C for 24 h. The assessment focused on sessile bacteria (adhering to the scaffold surface). Following incubation, the scaffolds were transferred to fresh wells, rinsed with sterile saline solution (0.9% NaCl) to remove non-adherent cells, and subsequently incubated for 1 h in a 10% resazurin solution prepared in TSB. The fluorescence intensity (excitation at 530 nm, emission at 590 nm) was recorded using a Synergy HT microplate reader (Biotek, Winooski, VT, USA) with Gen5 1.09 Data Analysis Software. The results were normalized against the untreated β-TCP scaffolds, which served as the control group.

#### 3.7.3. Antibacterial Indirect Assay

To examine the antimicrobial effect of the soluble factors released by the β-TCP scaffolds, extracts were obtained by incubating the samples in α-MEM medium for 24 h. The resulting extracts were subsequently diluted in TSB medium to final concentrations of 10%, 50%, and 90%. These diluted extracts were then used to challenge *S. aureus* in 96-well plates, with a final bacterial concentration of 5 × 10^5^ CFU/mL.

### 3.8. SEM Characterization of β-TCP Scaffolds Post-Biological Testing

β-TCP scaffolds, following adhesion of either hBM-MSCs or *S. aureus*, were initially fixed for 15 min in a 1.5% glutaraldehyde solution prepared in 0.1 M sodium cacodylate buffer (TAAB Laboratories Equipment Ltd., Aldermaston, UK) and subsequently stored in the same buffer. The samples underwent a stepwise dehydration process using ethanol solutions at increasing concentrations (50%, 70%, 90%, and 100%), followed by critical point drying (CPD 7501, Polaron Range).

To improve the imaging resolution, a thin (~4 nm) gold-palladium coating was applied via sputter coating. The morphological characteristics of the β-TCP scaffolds were then examined using SEM with a FEI Quanta 400 FEG ESEM/EDAX Genesis X4M system (FEI Company, Hillsboro, OR, USA).

### 3.9. Statistical Analysis

The results are presented with respect to the untreated samples, which were designated as the control group (control = 1.0). Data are expressed as the mean ± standard deviation (SD), and each experiment was performed in triplicate. To assess statistical significance, one-way ANOVA was applied, with the significance thresholds set at * *p* < 0.05, ** *p* < 0.01, *** *p* < 0.001, and **** *p* < 0.0001. All statistical analyses were conducted using GraphPad Prism version 9.0.0 (GraphPad Software, Inc., San Diego, CA, USA).

## 4. Conclusions

This study highlights that fs-laser-engineered β-TCP scaffolds functionalized with green-synthesized AgNPs surpass the ion-doped variants in achieving an optimal balance between antibacterial efficacy and osteogenic potential. The combination of fs-laser nanostructuring (4.1 J/cm^2^, linear pattern) and AgNPs, produced via an eco-friendly fs-laser photoreduction process using GTLE, demonstrated superior reductions in *S. aureus* viability. This enhanced antibacterial effect is attributed to the synergistic interplay of nanostructured surface disruption and AgNP-mediated bactericidal mechanisms. Importantly, AgNPs are hypothesized to remain surface-bound, facilitating contact-dependent bactericidal activity, while the absence of cytotoxicity may be partially explained by the protective effects of polyphenols present in GTLE.

In contrast, the ion-doped scaffolds exhibited limited direct antibacterial effects but showed concentration-dependent activity in the indirect assays, likely driven by controlled ion-release kinetics. However, higher fluences (6.1 J/cm^2^) and excessive doping were found to induce transient cytotoxicity, emphasizing the critical need for parameter optimization to ensure biocompatibility and bioactivity.

AgNP-functionalized scaffolds not only supported progressive metabolic activity in the hBM-MSCs but also promoted mineralized matrix deposition, as evidenced by the SEM morphologies suggestive of osteogenic differentiation. Furthermore, fs-laser-induced stabilization of the β-TCP/HA phase in doped scaffolds revealed a trade-off between structural integrity and bioactivity, highlighting the importance of balancing these properties in scaffold design.

Overall, this work identifies AgNP-functionalized fs-laser nanostructuring as the most effective strategy to achieve both robust pathogen resistance and enhanced osteointegration. By leveraging scalable and sustainable fabrication methods, this approach offers significant improvements in antibacterial efficacy and osteogenic compatibility, which are crucial for advancing bone regeneration therapies. To further support the biological findings, future work should include direct compositional analyses, such as X-ray photoelectron spectroscopy or inductively coupled plasma techniques, to confirm elemental retention and distribution on the scaffolds. This would strengthen the link between material properties and biological performance, guiding the optimization of multifunctional bone graft materials. To translate these promising findings into clinical applications, further research is essential to elucidate the underlying mechanisms and refine this approach for safe, effective use in bone-regenerative therapies and infection prevention.

## Figures and Tables

**Table 1:** Summary of the parameters used for developing non-doped and ion-doped β-TCP samples used for in vitro biological assays.

Scanning Velocity (mm/s)	Fluence (J/cm^2^)	Pattern	Doping
1	4.1	Linear	NA
1	4.1	Linear	AgNPs
1	4.1	Linear	Ions
1	6.1	Linear	NA
1	6.1	Linear	Ions
1	4.1	Crossed	NA
1	4.1	Crossed	Ions

## Data Availability

The original contributions presented in the study are included in the article; further inquiries can be directed to the corresponding authors.

## References

[1] Tande A.J., Patel R. (2014). Prosthetic Joint Infection. Clin. Microbiol. Rev..

[2] Zimmerli W., Trampuz A., Ochsner P.E. (2004). Prosthetic-Joint Infections. N. Engl. J. Med..

[3] Costerton J.W., Stewart P.S., Greenberg E.P. (1999). Bacterial Biofilms: A Common Cause of Persistent Infections. Science.

[4] Bohner M., Santoni B.L.G., Döbelin N. (2020). β-Tricalcium Phosphate for Bone Substitution: Synthesis and Properties. Acta Biomater..

[5] Clauss M., Trampuž A., Borens O., Bohner M., Ilchmann T. (2010). Biofilm Formation on Bone Grafts and Bone Graft Substitutes: Comparison of Different Materials by a Standard In Vitro Test and Microcalorimetry. Acta Biomater..

[6] Hameed S., Sharif S., Ovais M., Xiong H. (2024). Emerging Trends and Future Challenges of Advanced 2D Nanomaterials for Combating Bacterial Resistance. Bioact. Mater..

[7] Ketonis C., Parvizi J., Jones L.C. (2012). Evolving Strategies to Prevent Implant-Associated Infections. J. Am. Acad. Orthop. Surg..

[8] Zhang X., Zu H., Zhao D., Yang K., Tian S., Yu X., Lu F., Liu B., Yu X., Wang B. (2017). Ion Channel Functional Protein Kinase TRPM7 Regulates Mg Ions to Promote the Osteoinduction of Human Osteoblast via PI3K Pathway: In Vitro Simulation of the Bone-Repairing Effect of Mg-Based Alloy Implant. Acta Biomater..

[9] Mao L., Xia L., Chang J., Liu J., Jiang L., Wu C., Fang B. (2017). The Synergistic Effects of Sr and Si Bioactive Ions on Osteogenesis, Osteoclastogenesis and Angiogenesis for Osteoporotic Bone Regeneration. Acta Biomater..

[10] Baheiraei N., Eyni H., Bakhshi B., Najafloo R., Rabiee N. (2021). Effects of Strontium Ions with Potential Antibacterial Activity on In Vivo Bone Regeneration. Sci. Rep..

[11] Yan X., He B., Liu L., Qu G., Shi J., Hu L., Jiang G. (2018). Antibacterial Mechanism of Silver Nanoparticles in *Pseudomonas aeruginosa*: Proteomics Approach. Metallomics.

[12] Fang Z., Zhou Q., Zhang W., Wang J., Liu Y., Yu M., Qiu Y., Ma Z., Liu S. (2023). A Synergistic Antibacterial Study of Copper-Doped Polydopamine on Ti_3_C_2_T_x_ Nanosheets with Enhanced Photothermal and Fenton-like Activities. Materials.

[13] Long Y., Hu L., Yan X., Zhao X., Zhou Q., Cai Y., Jiang G. (2017). Surface Ligand Controls Silver Ion Release of Nanosilver and Its Antibacterial Activity against *Escherichia coli*. Int. J. Nanomed..

[14] Anees Ahmad S., Das S.S., Khatoon A., Ansari M., Afzal M., Hasnain S., Nayak A. (2020). Bactericidal Activity of Silver Nanoparticles: A Mechanistic Review. Mater. Sci. Energy Technol..

[15] Subha V., Ravindran E., Kumar A.B.H., Renganathan S. (2019). Bactericidal Effect of Silver Nanoparticles from Aqueous Root Extracts of *Catharanthus roseus*. Int. J. Nanoparticles.

[16] Yuan Y.-G., Peng Q.-L., Gurunathan S. (2017). Effects of Silver Nanoparticles on Multiple Drug-Resistant Strains of *Staphylococcus aureus* and *Pseudomonas aeruginosa* from Mastitis-Infected Goats: An Alternative Approach for Antimicrobial Therapy. Int. J. Mol. Sci..

[17] Kang J., Dietz M.J., Hughes K., Xing M., Li B. (2019). Silver Nanoparticles Present High Intracellular and Extracellular Killing against *Staphylococcus aureus*. J. Antimicrob. Chemother..

[18] Agnihotri S., Mukherji S., Mukherji S. (2014). Size-Controlled Silver Nanoparticles Synthesized over the Range 5–100 Nm Using the Same Protocol and Their Antibacterial Efficacy. RSC Adv..

[19] Agnihotri S., Mukherji S., Mukherji S. (2013). Immobilized Silver Nanoparticles Enhance Contact Killing and Show Highest Efficacy: Elucidation of the Mechanism of Bactericidal Action of Silver. Nanoscale.

[20] Kumari M., Shukla S., Pandey S., Giri V.P., Bhatia A., Tripathi T., Kakkar P., Nautiyal C.S., Mishra A. (2017). Enhanced Cellular Internalization: A Bactericidal Mechanism More Relative to Biogenic Nanoparticles than Chemical Counterparts. ACS Appl. Mater. Interfaces.

[21] Akter M., Sikder M.T., Rahman M.M., Ullah A.K.M.A., Hossain K.F.B., Banik S., Hosokawa T., Saito T., Kurasaki M. (2018). A Systematic Review on Silver Nanoparticles-Induced Cytotoxicity: Physicochemical Properties and Perspectives. J. Adv. Res..

[22] McNeilly O., Mann R., Hamidian M., Gunawan C. (2021). Emerging Concern for Silver Nanoparticle Resistance in *Acinetobacter baumannii* and Other Bacteria. Front. Microbiol..

[23] Oliver S., Wagh H., Liang Y., Yang S., Boyer C. (2018). Enhancing the Antimicrobial and Antibiofilm Effectiveness of Silver Nanoparticles Prepared by Green Synthesis. J. Mater. Chem. B.

[24] Oliveira M., Sousa A., Sá S., Soares S., Pereira A.C., Rocha A.C., Pais P., Ferreira D., Almeida C., Luís C. (2024). Harvesting the Power of Green Synthesis: Gold Nanoparticles Tailored for Prostate Cancer Therapy. Int. J. Mol. Sci..

[25] Palencia M.S., Berrio M.E., Palencia S.L. (2017). Effect of Capping Agent and Diffusivity of Different Silver Nanoparticles on Their Antibacterial Properties. J. Nanosci. Nanotechnol..

[26] Khansarizadeh M., Mokhtarzadeh A., Rashedinia M., Taghdisi S.M., Lari P., Abnous K.H., Ramezani M. (2016). Identification of Possible Cytotoxicity Mechanism of Polyethylenimine by Proteomics Analysis. Hum. Exp. Toxicol..

[27] Lan W.C., Lan W.H., Chan C.P., Hsieh C.C., Chang M.C., Jeng J.H. (1999). The Effects of Extracellular Citric Acid Acidosis on the Viability, Cellular Adhesion Capacity and Protein Synthesis of Cultured Human Gingival Fibroblasts. Aust. Dent. J..

[28] Huang H.-T., Cheng T.-L., Lin S.-Y., Ho C.-J., Chyu J.Y., Yang R.-S., Chen C.-H., Shen C.-L. (2020). Osteoprotective Roles of Green Tea Catechins. Antioxidants.

[29] Metwally S., Stachewicz U. (2019). Surface Potential and Charges Impact on Cell Responses on Biomaterials Interfaces for Medical Applications. Mater. Sci. Eng. C.

[30] Ferrari M., Cirisano F., Morán M.C. (2019). Mammalian Cell Behavior on Hydrophobic Substrates: Influence of Surface Properties. Colloids Interfaces.

[31] Filipov E., Angelova L., Vig S., Fernandes M.H., Moreau G., Lasgorceix M., Buchvarov I., Daskalova A. (2022). Investigating Potential Effects of Ultra-Short Laser-Textured Porous Poly-ε-Caprolactone Scaffolds on Bacterial Adhesion and Bone Cell Metabolism. Polymers.

[32] Luo X., Yao S., Zhang H., Cai M., Liu W., Pan R., Chen C., Wang X., Wang L., Zhong M. (2020). Biocompatible Nano-Ripples Structured Surfaces Induced by Femtosecond Laser to Rebel Bacterial Colonization and Biofilm Formation. Opt. Laser Technol..

[33] Gnilitskyi I., Rymar S., Iungin O., Vyshnevskyy O., Parisse P., Potters G., Zayats A.V., Moshynets O. (2023). Femtosecond Laser Modified Metal Surfaces Alter Biofilm Architecture and Reduce Bacterial Biofilm Formation. Nanoscale Adv..

[34] Siddiquie R.Y., Gaddam A., Agrawal A., Dimov S.S., Joshi S.S. (2020). Anti-Biofouling Properties of Femtosecond Laser-Induced Submicron Topographies on Elastomeric Surfaces. Langmuir.

[35] Carvalho A., Grenho L., Fernandes M.H., Daskalova A., Trifonov A., Buchvarov I., Monteiro F.J. (2020). Femtosecond Laser Microstructuring of Alumina Toughened Zirconia for Surface Functionalization of Dental Implants. Ceram. Int..

[36] Wu R., Li Y., Shen M., Yang X., Zhang L., Ke X., Yang G., Gao C., Gou Z., Xu S. (2021). Bone Tissue Regeneration: The Role of Finely Tuned Pore Architecture of Bioactive Scaffolds before Clinical Translation. Bioact. Mater..

[37] Xu S., Dou H., Sun K., Ye Y., Li Z., Wang H., Liao W., Liu H., Miao X., Yuan X. (2018). Scan Speed and Fluence Effects in Femtosecond Laser Induced Micro/Nano-Structures on the Surface of Fused Silica. J. Non-Cryst. Solids.

[38] Wang Y., Hu J., Zhang X., Chu Z., Ren B., Yue C., Jiang B., Liu X. (2023). Influence of Femtosecond Laser Pulse Sequence on the Morphology and Roughness of Titanium Surface Micro-Patterns. J. Manuf. Process..

[39] Qiu C., Panwisawas C., Ward M., Basoalto H.C., Brooks J.W., Attallah M.M. (2015). On the Role of Melt Flow into the Surface Structure and Porosity Development during Selective Laser Melting. Acta Mater..

[40] Sasidharan Pillai R., Sglavo V.M. (2014). Effect of MgO Addition on Solid State Synthesis and Thermal Behavior of Beta-Tricalcium Phosphate. Ceram. Int..

[41] Kliuev M., Wiessner M., Büttner H., Maradia U., Wegener K. (2020). Super-Hydrophobic and Super-Hydrophilic Effect by Means of EDM Surface Structuring of γ-TiAl. Procedia CIRP.

[42] Arahira T., Maruta M., Matsuya S. (2017). Characterization and In Vitro Evaluation of Biphasic α-Tricalcium Phosphate/β-Tricalcium Phosphate Cement. Mater. Sci. Eng. C.

[43] Wang J., Qian J., Xu W., Wang Y., Hou G., Sun T., Luo L. (2017). Effects of Sr^2+/^Zn^2+^ Co-Substitution on Crystal Structure and Properties of Nano-β-Tricalcium Phosphate. Ceram. Int..

[44] Zhang X., Jiang F., Groth T., Vecchio K.S. (2008). Preparation, Characterization and Mechanical Performance of Dense β-TCP Ceramics With/without Magnesium Substitution. J. Mater. Sci. Mater. Med..

[45] Frasnelli M., Sglavo V.M. (2016). Effect of Mg^2+^ Doping on Beta–Alpha Phase Transition in Tricalcium Phosphate (TCP) Bioceramics. Acta Biomater..

[46] Banerjee S.S., Tarafder S., Davies N.M., Bandyopadhyay A., Bose S. (2010). Understanding the Influence of MgO and SrO Binary Doping on the Mechanical and Biological Properties of β-TCP Ceramics. Acta Biomater..

[47] Shuai C., Zhuang J., Peng S., Wen X. (2014). Inhibition of Phase Transformation from β- to α-Tricalcium Phosphate with Addition of Poly (L-Lactic Acid) in Selective Laser Sintering. Rapid Prototyp. J..

[48] Somers N., Jean F., Lasgorceix M., Curto H., Urruth G., Thuault A., Petit F., Leriche A. (2021). Influence of Dopants on Thermal Stability and Densification of β-Tricalcium Phosphate Powders. Open Ceram..

[49] Shaikh S., Kedia S., Singh A.K., Sharma K., Sinha S. (2016). Surface Treatment of 45S5 Bio-Glass Using Femtosecond Laser to Achieve Superior Growth of Hydroxyapatite. arXiv.

[50] Dautova Y., Kozlova D., Skepper J.N., Epple M., Bootman M.D., Proudfoot D. (2014). Fetuin-A and Albumin Alter Cytotoxic Effects of Calcium Phosphate Nanoparticles on Human Vascular Smooth Muscle Cells. PLoS ONE.

[51] Motskin M., Wright D.M., Muller K., Kyle N., Gard T.G., Porter A.E., Skepper J.N. (2009). Hydroxyapatite Nano and Microparticles: Correlation of Particle Properties with Cytotoxicity and Biostability. Biomaterials.

[52] Verberckmoes S.C., De Broe M.E., D’Haese P.C. (2003). Dose-Dependent Effects of Strontium on Osteoblast Function and Mineralization. Kidney Int..

[53] Leidi M., Dellera F., Mariotti M., Maier J.A.M. (2011). High Magnesium Inhibits Human Osteoblast Differentiation In Vitro. Magnes. Res..

[54] Zhang L., Yang C., Li J., Zhu Y., Zhang X. (2014). High Extracellular Magnesium Inhibits Mineralized Matrix Deposition and Modulates Intracellular Calcium Signaling in Human Bone Marrow-Derived Mesenchymal Stem Cells. Biochem. Biophys. Res. Commun..

[55] Rest J.R. (1976). The Histological Effects of Copper and Zinc on Chick Embryo Skeletal Tissues in Organ Culture. Br. J. Nutr..

[56] Murni N.S., Dambatta M.S., Yeap S.K., Froemming G.R.A., Hermawan H. (2015). Cytotoxicity Evaluation of Biodegradable Zn–3Mg Alloy toward Normal Human Osteoblast Cells. Mater. Sci. Eng. C.

[57] Mathew S., Abraham T.E., Zakaria Z.A. (2015). Reactivity of Phenolic Compounds towards Free Radicals under In Vitro Conditions. J. Food Sci. Technol..

[58] Gérard C., Goldbeter A. (2014). The Balance between Cell Cycle Arrest and Cell Proliferation: Control by the Extracellular Matrix and by Contact Inhibition. Interface Focus.

[59] Ribatti D. (2017). A Revisited Concept: Contact Inhibition of Growth. From Cell Biology to Malignancy. Exp. Cell Res..

[60] Bispo D.S.C., Jesus C.S.H., Correia M., Ferreira F., Bonifazio G., Goodfellow B.J., Oliveira M.B., Mano J.F., Gil A.M. (2022). NMR Metabolomics Assessment of Osteogenic Differentiation of Adipose-Tissue-Derived Mesenchymal Stem Cells. J. Proteome Res..

[61] Matta C., Szűcs-Somogyi C., Kon E., Robinson D., Neufeld T., Altschuler N., Berta A., Hangody L., Veréb Z., Zákány R. (2019). Osteogenic Differentiation of Human Bone Marrow-Derived Mesenchymal Stem Cells Is Enhanced by an Aragonite Scaffold. Differentiation.

[62] Huang H., Zhang P., Yu Z., Zhang X., Shen L., Shi H., Yan H., Wang L., Tian Y. (2022). Effects of Periodic Surface Structures Induced by Femtosecond Laser Irradiation on the Antibacterial Properties of Zr-Based Amorphous Material. Optik.

[63] Zhang Y., Pan X., Liao S., Jiang C., Wang L., Tang Y., Wu G., Dai G., Chen L. (2020). Quantitative Proteomics Reveals the Mechanism of Silver Nanoparticles against Multidrug-Resistant *Pseudomonas aeruginosa* Biofilms. J. Proteome Res..

[64] Li W.-R., Xie X.-B., Shi Q.-S., Duan S.-S., Ouyang Y.-S., Chen Y.-B. (2010). Antibacterial Effect of Silver Nanoparticles on *Staphylococcus aureus*. BioMetals.

[65] Mirzajani F., Askari H., Hamzelou S., Schober Y., Römpp A., Ghassempour A., Spengler B. (2014). Proteomics Study of Silver Nanoparticles Toxicity on *Bacillus thuringiensis*. Ecotoxicol. Environ. Saf..

[66] De Rossi L., Rocchetti G., Lucini L., Rebecchi A. (2025). Antimicrobial Potential of Polyphenols: Mechanisms of Action and Microbial Responses—A Narrative Review. Antioxidants.

[67] Ke D., Tarafder S., Vahabzadeh S., Bose S. (2019). Effects of MgO, ZnO, SrO, and SiO_2_ in Tricalcium Phosphate Scaffolds on In Vitro Gene Expression and In Vivo Osteogenesis. Mater. Sci. Eng. C.

[68] (1997). Geometrical Product Specifications (GPS)—Surface Texture: Profile Method—Terms, Definitions and Surface Texture Parameters.

[69] Sun Q., Cai X., Li J., Zheng M., Chen Z., Yu C.-P. (2014). Green Synthesis of Silver Nanoparticles Using Tea Leaf Extract and Evaluation of Their Stability and Antibacterial Activity. Colloids Surf. A Physicochem. Eng. Asp..

[70] Oliveira M., Angelova L., Grenho L., Fernandes M.H., Daskalova A. (2024). Dual-Function Femtosecond Laser: β-TCP Structuring and AgNP Synthesis via Photoreduction with Azorean Green Tea for Enhanced Osteointegration and Antibacterial Properties. Materials.

[71] (2018). Biological Evaluation of Medical Devices.

[72] (2012). Staphylococcus aureus, ATCC^®^ 25923^TM^.

